# The Treatment of Advanced Cardiac Dilatation

**Published:** 1905-05-06

**Authors:** 


### THE TREATMENT OF ADVANCED CARDIAC DILATATION.

In a large majority of young adults who suffer from cardiac dilatation as a secondary result of rheumatic endocarditis the myocardium is in a sufficiently good state to permit of a rapid arrest of the dilatation by means of rest and the established medicinal treatment by digitalis or strophanthus. But after middle age the condition of the heart's muscle becomes more and more problematical, and the response to treatment progressively less certain and less complete. And when digitalis and rest fail to induce the return of a dilated heart to its proper proportions and functions it must be admitted that treatment seems to be approaching an *impasse*. Dr. R. A. Fleming[1] has published a plea for massage, followed by resistance movements, and ultimately by graduated exercise, as a useful adjunct to the accepted principles of treatment in these very difficult cases. He finds that most authors are agreed that massage has a favourable influence upon the heart, though the theoretical explanations of this influence are widely different. There seems to be no mystery about the method, no particular movements are ordered, and no special instructions are given, except that the movements are limited to such actions of the arms and legs as can be carried out in bed, and that the limit of fatigue is to be carefully avoided.

The type of case to which the author refers, the details of the treatment, and the result, are exemplified in the following one, taken from his series.

A woman, aged 47, in June 1903 took a very hot bath, and remained in it for a long time; the next day she took a longer and more vigorous walk than was her custom, and on the following day became faint and breathless, and suffered from palpitation and giddiness. On examination her heart was found to be greatly dilated; at the level of the fourth rib the cardiac dulness extended 2¼ inches to the right of the mid-sternal line, and seven inches to the left of this line. The apex beat, which to the author's knowledge had a few months before been situated under the sixth rib, was now under the seventh rib, and in the anterior axillary line. The pulse intermitted every three or four beats, and was very irregular in force. There were systolic murmurs in the mitral and tricuspid areas, and signs of œdema at the pulmonary bases. The ankles also were œdematous.

After long treatment by rest in bed and the administration of strophanthus she began to improve, but on two occasions relapsed after attempting to lift a box. After the second relapse she was in bed for six months; the slightest movement was sufficient to bring on symptoms of heart failure, and for 11 months practically no advance was made. It has been necessary to set the case out at some length to show that it really was a very intractable one up to this time. But at this point massage was commenced and carried on for ten days; on the eleventh day resisted movements were cautiously attempted and carried on daily for a second period of ten days; on the twenty-first day the patient was allowed to get up for half an hour, and a five-minute period of gentle walking was substituted for the resisted exercises. Under this treatment she slowly but steadily improved; the pulse became more regular and slower in rate, and she ate and slept much better; the heart returned to its normal size and the persistent cough disappeared. Eight months later compensation was sufficiently established to admit of the patient's being up all day, of her taking moderate exercise, and doing light forms of work such as sewing. During the treatment all medication was discontinued, with the exception that a small dose of strophanthus was given as a precaution when the systematic exercises ceased to be carried out.

Dr. Fleming states that in one case only of the ten upon which his experience of the treatment is based, did any harm result from the procedure, and concludes his opinion in the matter as follows: "Even eight successes, or moderate successes, out of ten cases so treated seem to me to warrant the systematic trial of the method. In over half the cases considerable periods have elapsed without any break-down of compensation, periods varying from two years to six months."

[1] Scottish Med. and Surg. Jour., March 1905.

